# FOXM1 Promotes Head and Neck Squamous Cell Carcinoma *via* Activation of the Linc-ROR/LMO4/AKT/PI3K Axis

**DOI:** 10.3389/fonc.2021.658712

**Published:** 2021-08-10

**Authors:** Xiao Ma, Hong Zhang, Qian Li, Erik Schiferle, Yao Qin, Suifang Xiao, Tiancheng Li

**Affiliations:** ^1^Department of Head and Neck Surgery, Perking University Cancer Hospital and Institute, Beijing, China; ^2^Department of Pathology, Peking University First Hospital, Beijing, China; ^3^Department of Radiology, Massachusetts General Hospital, Boston, MA, United States; ^4^Center for Cancer Research, Massachusetts General Hospital and Harvard Medical School, Boston, MA, United States; ^5^Department of Otorhinolaryngology-Head and Neck Surgery, Peking University First Hospital, Beijing, China

**Keywords:** head and neck squamous cell carcinoma, FoxM1, linc-ROR, LMO4, Akt/PI3K, proliferation, invasion

## Abstract

**Background/Aim:**

Previous literature has implicated the sustained expression of FOXM1 in numerous human cancers, including head and neck squamous cell carcinoma (HNSCC). The current study aimed to elucidate the function and regulatory mechanism of FOXM1 in HNSCC.

**Methods:**

Western blot and RT-qPCR methods were performed to evaluate the expression of Linc-ROR, FOXM1, and LMO4 in HNSCC tissue samples and cells. The binding between FOXM1 and Linc-ROR was analyzed using a ChIP assay. Various cellular processes including proliferation and invasion abilities were assessed following alteration of FOXM1, Linc-ROR and LMO4 expression in HNSCC cells. Xenograft mouse models were established to validate the *in vitro* findings.

**Results:**

Linc-ROR and FOXM1 were highly expressed in HNSCC tissues and cells. FOXM1 operated as a potential transcription factor to bind to the promoter region of Linc-ROR. Linc-ROR and FOXM1 exhibited high expression levels in both the clinical tissue samples as well as the HNSCC cells, which could facilitate the proliferation and invasion of HNSCC cells. Linc-ROR upregulated the expression of LMO4 and promoted activation of the AKT/PI3K signaling pathway, thus stimulating the proliferation and invasion of HNSCC cells. Silencing of Linc-ROR brought about a contrasting effect relative to that seen when FOXM1 was overexpressed in HNSCC *in vivo*.

**Conclusions:**

Overall, FOXM1 promoted the expression of Linc-ROR and induced the activation of the LMO4-dependent AKT/PI3K signaling pathway, thus facilitating the occurrence and development of HNSCC.

## Introduction

Head and neck squamous cell carcinoma (HNSCC) represents a malignancy encompassing a heterogeneous population of cancer cells that generally arise from the squamous epithelium of the oral cavity, oropharynx, larynx, as well as hypopharynx (1). HNSCC can be divided into two distinct subgroups, human papillomavirus (HPV)-positive HNSCC and HPV-negative HNSCC, based on patient HPV infection history (1). HPV-positive HNSCC is typically seen in young adults and non-smokers (2). HNSCC has been strongly associated with tobacco smoking (3). Studies have indicated that the annual incidence of HNSCC is approximately 800,000 new cases globally (4). In addition to surgical management (5), chemotherapy remains the first-line approach for advanced cases of HNSCC, with agents such as platinum combined with cetuximab, nivolumab or pembrolizumab often employed (6, 7). Despite clinical success of the aforementioned modalities, the five-year survival rate remains unsatisfactory (8).

As a nuclear transcription factor regulating cell cycle genes, the sustained expression of forkhead box M1 (FOXM1) has been highlighted as a hallmark of nearly all human cancers (9). The epigenetic signature induced by FOXM1 has been speculated to possess the clinical translational potential to function as a biomarker for HNSCC, including early cancer screening, diagnosis, and therapy, as it can mimic the cancer epigenome (10). Moreover, the knockdown of FOXM1 has been reported to encumber the proliferation and invasion of HNSCC cells, along with a more differentiated-like phenotype in three-dimensional culture and Xenograft tumor models (11). In hepatocellular carcinoma cell lines, FOXM1 was previously observed to transcriptionally activate long intergenic non-protein coding RNA regulator of reprogramming (Linc-ROR) (12). Located on 18q21.31, Linc-ROR has been reported to contribute to tumor growth by means of regulating the reprogramming of plu ripotent stem cells (13). Interestingly, the over-expression of Linc-ROR has also been emphasized in undifferentiated tumors, with studies suggesting the existence of a strong correlation with tumor recurrence and poor response to treatment (14). Certain lncRNAs have been reported to facilitate cancer progression by positively regulating LIM-only protein 4 (LMO4) (15). LMO4 is a nuclear adapter that is a pivotal factor for the assembly of multi-protein transcriptional complexes and its dysregulated expression is characteristic of a number of epithelial malignancies, including that of HNSCC (16). LMO4 possesses the ability to promote tumor cell migration and invasion in non-small-cell lung cancer *via* the involvement of the protein kinase B/phosphatidylinositol 3-kinase (AKT/PI3K) signaling pathway (17). The AKT/PI3K signaling pathway often exhibits hyper activation in numerous types of cancers (18). Moreover, the enhanced activity of AKT/PI3K signaling pathway, which is often altered in HPV-positive and negative HNSCC, has been linked with poor patient prognosis (19). Herein, we hypothesized that FOXM1 might participate with Linc-ROR, LMO4 and the AKT/PI3K signaling pathway in the occurrence and progression of HNSCC. We subsequently performed a series of experiments to explore and determine this hypothesis.

## Methods

### Ethics Statement

The current study was performed with the approval of the Ethics Committee of Peking University First Hospital, and conducted following the *Declaration of Helsinki*. All participants signed written informed consent documentation prior to enrollment. All animal experiments were approved by the Institutional Animal Care and Use Committee of Peking University First Hospital. Extensive efforts were made to ensure minimal suffering of the included animals.

### Bioinformatics Analysis

RNA-seq expression profiles of HNSCC were obtained from the TCGA database (https://xenabrowser.net/datapages/), including 502 HNSCC samples and 44 normal samples. R package limma was applied to analyze the expression of Linc-ROR and FOXM1 in both HNSCC samples as well as the normal samples. ChIP-seq data from ENCOD TFBS were explored and analyzed in the UCSC database (https://genome-asia.ucsc.edu/index.html) to predict transcription factors for Linc-ROR.

### Clinical Sample Collection

Thirty-four patients with HNSCC admitted to Peking University First Hospital from January 2016 to March 2019 were recruited for the purposes of the current study. The HNSCC biopsy specimens and adjacent normal tissues were collected. Briefly, the tissues were surgically resected, promptly flash-frozen in liquid nitrogen and subsequently stored at -80°C. The age of the patients ranged from 29 to 73 years, with all patients confirmed to have not undergone any chemotherapy or radiotherapy treatment prior to surgery. Pathologic characteristics examined included age, sex, etiology, AFP, tumor size, tumor differentiation, and stage. Tumors were classified and graded based on the pathological tumor-node-metastasis (pTNM) staging advocated by the International League (20).

### Cell Culture and Lentivirus Transfection

Human tongue squamous carcinoma cell line (TSCCA, from the website of microorganism for search, http://www.biobw.org/), normal oral mucosa epithelial cell line (HNOEC/HL-047, from the American Type Culture Collection Repository [ATCC], Manassas, VA, USA), and human embryonic kidney cell line (293T, from ATCC) were cultured in Dulbecco’s modified Eagle’s medium (DMEM, Thermo Fisher Scientific Inc., Waltham, MA, USA) supplemented with 10% fetal bovine serum (FBS, Gibco, Carlsbad, CA, USA) in an incubator at 37°C with 5% CO_2_. Transfection of Linc-ROR, FOXM1, LMO4, negative control shRNAs, and overexpression lentivirus (pSIH1-H1-copGFP) were purchased from GenePharma (Shanghai, China). The HEK293T cells were subsequently co-transfected with packaging virus and target vector, with the supernatant collected following 48 hours of cell culture. Viruses in the logarithmic growth phase were collected and divided into 1) control group, 2) short hairpin RNA-negative control (sh-NC) group, 3) sh-Linc-ROR-1 group, 4) sh-Linc-ROR-2 group, 5) sh-FOXM1-1 group, 6) sh-FOXM1-2 group, 7) sh-LMO4-1 group, 8) sh-LMO4-2 group, 9) overexpression (oe)-NC group, 10) oe-FOXM1 group, 11) oe-Linc-ROR group, 12) oe-LMO4 group, 13) sh-Linc-ROR + oe-LMO4 group, and 14) oe-FOXM1 + sh-Linc-ROR group. Cells were cultured in 6-well plates for transfection. After 48 h of infection, the expression of the related genes in each group of cells was detected using reverse transcription quantitative polymerase chain reaction (RT-qPCR). Each experiment was performed in triplicate.

### RT-qPCR

Total RNA was isolated from cultured cells by TRIzol reagents (15596-018, Solarbio, Beijing, China), while first-strand complementary DNA synthesis was performed using a cDNA Reverse Transcript kit (Reanta, Beijing, China). The cDNA levels were then amplified using the ViiA 7 Real-Time PCR detection system (DaanGene, Guangzhou, China). The primers for the qPCR reactions were synthesized by Takara (Kusatsu, Japan) and listed in **Table S1**. Gene expression was determined in triplicate and normalized to glyceraldehyde-3-phosphate dehydrogenase (GAPDH). The relative expression of each target gene was calculated using the 2^-ΔΔCT^ method. Each experiment was performed in triplicate.

### Western Blot

Total protein was extracted from cells or tissues using high-performance RIPA lysis buffer (R0010, Solarbio), with the concentration determined using a bicinchoninic acid (BCA) assay kit (Yeasen, Shanghai, China). The proteins from each sample were then separated *via* sodium dodecyl sulfate-polyacrylamide gel electrophoresis and transferred onto a polyvinylidene fluoride membrane (Millipore, Billerica, MA, USA) *via* the wet-transfer method. The membrane was blocked with 5% bovine serum albumin for 1 h at room temperature and incubated with primary antibodies against FOXM1 (ab180710, 1:1000, Abcam, Cambridge, UK), LMO4 (ab131030, 1:1000, Abcam), AKT (ab8805, 1:500, Abcam), PI3K (ab40776, 1:1000, Abcam), or phosphorylated (p)-PI3K (ab182651, 1:1000, Abcam), followed by incubation with horseradish peroxidase (HRP)-conjugated secondary antibody (ab205718, 1:10000, Abcam) at room temperature for 1 h. Development was performed using VILBER FUSION FX5 (Vilber Lourmat, France). The protein bands were quantified using ImageJ 1.48 (National Institute of Health, Bethesda, MD, USA), and normalized to GAPDH. Each experiment was performed in triplicate.

### Immunohistochemical (IHC) Staining

The HNSCC tissues were fixed in 4% paraformaldehyde for 12 h, cleared with xylene, and rehydrated using 100% ethanol, 95% ethanol and 75% ethanol. The tissues were then boiled in 0.01 M citrate buffer for 15-20 min, washed with phosphate-buffered saline (PBS), blocked with goat serum solution for 20 min followed by incubation with anti-FOXM1 antibody (ab180710, 1:200, Abcam) or anti-LMO4 antibody (ab229226, 1:100, Abcam) for 1 hour at room temperature. After rinsing in PBS, the tissue sections were incubated with SP-conjugated sheep anti-rabbit IgG for 1 h at room temperature followed by PBS washing. The reaction was terminated by washing with tap water for 10 min followed by 5-10 min of diaminobenzidine (DAB) development. The tissue sections were subsequently stained with hematoxylin for 2 min and hydrochloric acid alcohol, followed by washing with tap water for 10 min. After dehydration, transparency and mounting were performed, with the tissue sections analyzed under a bright-field Olympus BX-60 microscope.

### *In Situ* Hybridization (ISH)

In situ hybridization was performed based on (21). Paraffin-embedded sections of tissues from HNSCC patients were deparaffinized and rehydrated. The sections were quenched for endogenous peroxidase activity purposes using 3% H_2_O_2_ for 30 min. After digestion with proteinase K for 5 min, the sections were fixed in 4% paraformaldehyde, rinsed in PBS, incubated in a hybridization buffer for 2 h at 60°C, then with Linc-ROR or scrambled control probe (50 nM digitoxin-labeled LNA probe, Exiqon, Vedbaek, Denmark) overnight at 60°C, and rinsed with washing buffer (50% formamide in 2× SSC and phosphate-buffered saline-Tween-20 [PBST]). Nitrotetrazolium was used for alkaline phosphate reactions. The sections were then sealed in a water-based mounting agent. Three fields of view were randomly selected and captured from each sample using a bright-field Olympus BX-60 microscope.

### Xenograft Tumor in Nude Mice

BALA/c nude mice (4 weeks, 18-25 g), purchased from the SJA laboratory Animal (Hunan, China), were housed and maintained in a SPF environment. All mice were randomly separated into 5 groups: 1) oe-NC group (n = 15), 2) oe-FOXM1 group (n = 15), 3) sh-NC group (n = 15), 4) sh-Linc-ROR group (n = 15), and 5) oe-FOXM1 + sh-LincROR group (n = 15), respectively injected with 5 × 10^5^ preconditioned TSCCA cells corresponding to the group. Volume measurement of the tumor was performed at the start of the 3^rd^ day after injection, based on the following formula: V = (A × B^2^)/2 (A is the long diameter, B is the short diameter). At the end of the experiment, the mice were euthanized *via* carbon dioxide inhalation. A curve depicting the average volume at each time point was subsequently plotted.

### Terminal Deoxynucleotidyl Transferase-Mediated dUTP-Biotin Nick End-Labeling (TUNEL) Staining

TUNEL was performed to detect the apoptosis of the nude mice. TUNEL staining was performed after the fixed-tissues (4% paraformaldehyde, overnight) had been embedded in paraffin and sectioned at a thickness of 5 mm. Five sections of the nude mouse tissues were dewaxed, followed by the addition of 50 μL of 1% proteinase K diluent, and incubated at 37°C for 30 min. The endogenous POD activity was then eliminated using 0.3% H_2_O_2_ methanol solution at 37°C for 30 min. The TUNEL reaction solution was added and incubated under dark conditions at 37°C for 1 h. Converter-POD was used at 37°C for 30 min. Finally, 2% DAB chromogenic solution was employed at room temperature for 15 min and observed under a OLYMPUS-CX43 microscope. In the event that brownish-yellow nuclei were observed, the reaction was terminated by distilled water. After hematoxylin counterstaining, and dehydration by gradient ethanol (50, 70, 90 and 100%), the sections were analyzed using a microscope. Ten fields were randomly taken from each section. The nuclei of the apoptotic cells were dyed brownish-yellow, while the nuclei of normal cells were dyed blue. The numbers of apoptosis cells in each field of view were calculated based on average findings.

### Plate Colony Formation Assay

A monoclonal formation assay was employed to detect the proliferation of cells as well as different groups of TSCCA cells. The control cells at the logarithmic phase were digested with trypsin and counted. Using a 6-well plate, 1 × 10^3^ TSCCA cells were seeded into each well with 2 mL DMEM with 10% FBS. After 7-10 days, the resulting colonies were fixed with methanol at room temperature for 15 min and subsequently stained with crystal violet. The colonies were then counted using a microscope (CX43, OLYMPUS, Japan). Each experiment was performed in triplicate.

### 3-(4,5-dimethylthiazol-2-yl)-2, 5-diphenyltetrazolium Bromide (MTT) Assay

MTT kit (C0009, Beyotime, Shanghai, China) was used to explore the proliferation of the TSCCA cells. After 24 h of transfection, 5 × 10^3^ cells were seeded into 96-well plates in triplicate and cultured in DMEM containing 10% FBS. MTT reagent (5 mg/mL) was then added (20 μL to each well), after which the plates were incubated for 4 h. The absorbance of the supernatant was measured using a microplate reader (US6111636, Thermo Fisher Scientific) at 490 nm. The results were plotted as the average absorbance of each group/fold of control value.

### Transwell Assay

ECM Matrigel (E1270-1ML, Sigma-Aldrich, St. Louis, MO, USA) was placed at 4°C overnight and diluted at a ratio of 1:9 with serum-free medium to a final concentration of 1 mg/mL. The diluted ECM (40 μL) was added to the polycarbonate membrane in the upper chamber of 24-well Transwell plate and incubated in 5% CO_2_, 37°C for 5 h. After ECM polymerized, the excess liquid was removed followed by the addition of 70 μL DMEM for incubation purposes in 5% CO_2_, 37°C for 0.5 h to rehydrate the Matrigel. Culturing in serum-free medium for 24 h, the TSCCA cells were resuspended in DMEM without FBS and seeded to the upper chamber of Transwell inserts. DMEM containing 10% FBS was added to the lower chamber. Following incubation for 24 h at 37°C, 5% CO_2_, the cells on the inserts and membranes were wiped off, and the remaining cells were fixed with methanol for 30 min and stained with 0.1% crystal violet staining solution for 20 min. Five fields were randomly selected under an inverted microscope (CKX41, Olympus, Tokyo, Japan) and the number of cells passing through the membrane was counted and averaged.

### Cytoplasmic/Nuclear Fractionation Assay

Nuclear and cytoplasmic RNA or proteins were isolated with a nucleocytoplasmic isolation kit (K266-25, Biovision, San Francisco, CA, USA). The cells were washed twice with PBS (on ice) and then centrifuged at 500 ×g for 5 min. The cells were subsequently resuspended in cell fractionation buffer, incubated for 10 min (on ice) and centrifuged at 500 ×g for 5 min. The supernatant was then applied for purification of cytoplasmic RNA or protein. Nuclear fraction was washed with cell fractionation buffer and homogenized with cell disruption buffer (12).

### Chromatin Immunoprecipitation (ChIP) Assay

The TSCCA cells were fixed with formaldehyde for 10 min to yield DNA-protein cross-linking, while an ultrasound disruptor (UP-250, Scientzbio, Ningbo, China) was used to break the cells and interrupt the chromatin into fragments (10 s ultrasound with 10 s interval, and cycled 15 times). The supernatant was then collected *via* centrifugation at 12000 rpm for 10 min, and divided into two tubes, which were incubated with NC rabbit IgG (ab1098489, 1:300, Abcam) or FOXM1 antibody (ab180710, 1:100, Abcam) overnight at 4°C. The DNA-protein complexes were purified with Agarose/Sepharose overnight at 65°C, and centrifuged at 12,000 g for 5 min in order to remove the supernatants. The non-specific complex was then washed, and the cross-linking was decrosslinked overnight at 65°C. The DNA fragments were extracted and purified with phenol/chloroform. The FOXM1 binding to Linc-ROR was detected by RT-qPCR using Linc-ROR-specific primers.

### Dual Luciferase Reporter Assay

The 3’ untranslated regions (UTR) dual-luciferase reporter plasmid of FOXM1 and mutant plasmids mutated with Linc-ROR binding site (PmirGLO-linc-ROR-WT and PmirGLO-linc-ROR-MUT) were constructed, respectively. The oe-ROXM1 plasmid or negative control plasmid was subsequently co-transfected into HEK293T cells. After transfection for 24 h, the cells were lysed and centrifuged. The supernatant was then collected after which the luciferase activity was measured using a Dual-Luciferase Reporter Assay System (E1910, Promega, Madison, WI, USA). Each experiment was performed in triplicate (12).

### Statistical Analysis

All data was analyzed using SPSS 21.0 software (IBM Corp. Armonk, NY, USA). Measurement data were expressed as the mean ± standard deviation. Paired *t*-test methods were applied for data comparison between cancer and adjacent normal tissues. Unpaired *t*-test methods were used for data comparison between two groups. Tukey’s test-corrected one-way analysis of variance (ANOVA) was applied during data comparison between multiple groups. Variables were analyzed at different time points using Bonferroni-corrected repeated measure ANOVA. *p <* 0.05 was considered to be indicative of statistical significance.

## Results

### FOXM1 and Linc-ROR Are Highly Expressed in Clinical Tissues of HNSCC Patients

**Figure 1:**
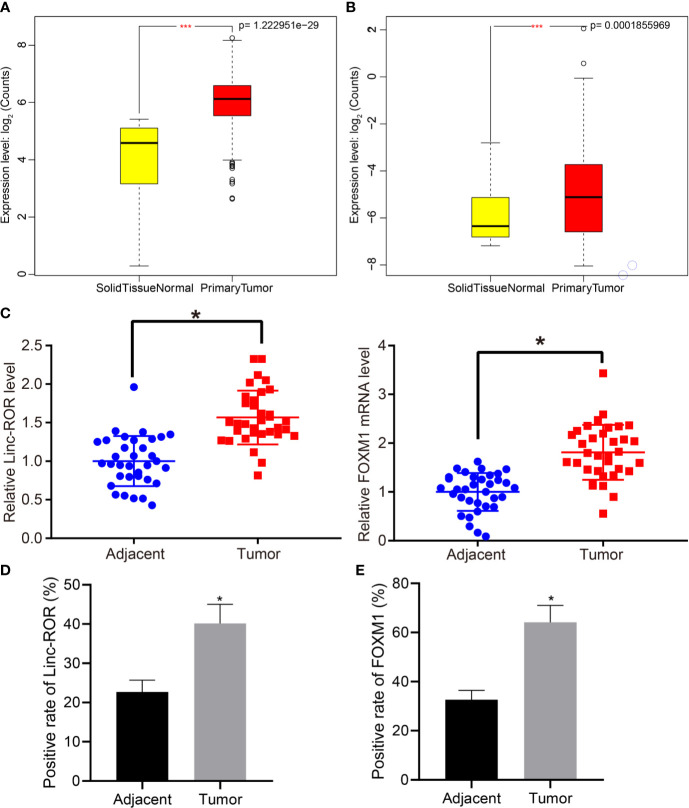
FOXM1 and Linc-ROR are highly expressed in clinical samples of HNSCC patients. **(A)** Expression of Linc-ROR of HNSCC samples in TCGA database; **(B)** Expression of FOXM1 in HNSCC samples in TCGA database; in **(A, B)**, the abscissa axis indicates sample grouping, ordinate axis indicates gene expression count after log2 value; N = 546, *** indicates *p <* 0.001. **(C)** RT-qPCR detection of Linc-ROR and FOXM1 expression in HNSCC and adjacent normal tissues of 34 HNSCC patients; **(D)** Expression of Linc-ROR in cancer and adjacent normal tissues of 34 HNSCC patients determined by in *situ* hybridization; **(E)** Expression of FOXM1 in cancer and adjacent normal tissues of 34 HNSCC patients determined by IHC. *p < 0.05, compared with adjacent normal tissues.

Previous literature has suggested that Linc-ROR induces histone methylation to promote tumorigenesis (22, 23). A previous study presented evidence indicating that FOXM1 could regulate drug resistance in head and neck cancer cells through Linc-ROR (23). The differential analysis of the 546 HNSCC data in TCGA revealed a high expression of Linc-ROR and FOXM1 in HNSCC samples (**Figures 1A, B**). Thus, we asserted the hypothesis that Linc-ROR and FOXM1 could promote the progression of HNSCC. We initially detected the expression of FOXM1 and Linc-ROR in HNSCC and adjacent normal tissues from 34 HNSCC patients *via* RT-qPCR, with the results revealing that Linc-ROR and FOXM1 were highly expressed in HNSCC tissues when compared to that of the adjacent normal tissues (**Figures 1C**). The ISH and IHC results revealed markedly higher expression levels of Linc-ROR and FOXM1 in the HNSCC tissues when compared with that in the adjacent normal tissues (**Figures 1D, E**). The aforementioned findings demonstrated that Linc-ROR and FOXM1 were highly expressed in HNSCC.

### Linc-ROR and FOXM1 Are Highly Expressed in HNSCC Cells and Promote HNSCC Cell Proliferation and Invasion

**Figure 2:**
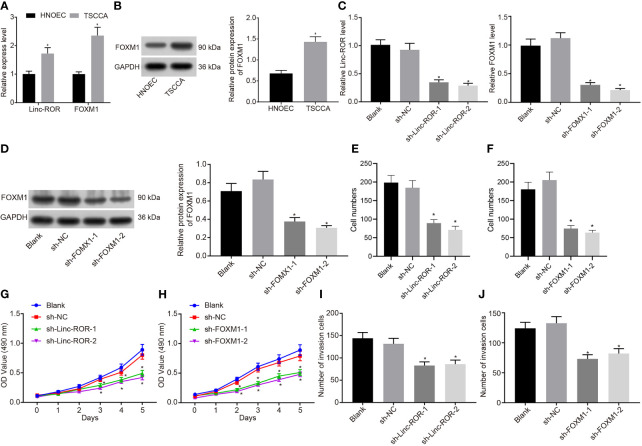
Linc-ROR and FOXM1 are highly expressed in HNSCC cells and promote cell proliferation and invasion. **(A)** The expression of Linc-ROR and FOXM1 in HNOEC/HL-047 and TSCCA cells detected by RT-qPCR; **(B)** The protein expression of FOXM1 in HNOEC/HL-047 and TSCCA cells detected by Western blot; **(C)** The knockdown effects of Linc-ROR and FOXM1 detected by RT-qPCR; **(D)** The knockdown effects of FOXM1 detected by Western blot; **(E)** Proliferation ability of TSCCA cells after silencing Linc-ROR detected by monoclonal formation assay; **(F)** Proliferation ability of TSCCA cells after silencing FOXM1 by monoclonal formation assay; **(G)** Proliferation ability of TSCCA cells after silencing Linc-ROR detected by MTT assay; **(H)** Proliferation ability of TSCCA cells after silencing FOXM1 detected by MTT assay; **(I)** Invasion capacity of TSCCA cells after silencing Linc-ROR detected by Transwell assay; **(J)** Invasion capacity of TSCCA cells after silencing FOXM1 detected by Transwell assay. In panel **(A, B)**, *p < 0.05, compared with the HNOEC/HL-047 cells, in panel **(C–J)**, TSCCA cells transfected with sh-NC. All experiments were repeated three times.

Next, to further determine whether Linc-ROR and FOXM1 influence the proliferation and invasion of HNSCC cells, we detected the expression of Linc-ROR and FOXM1 in HNSCC cell line TSCCA and the normal oral mucosal epithelial cell line (HNOEC/HL-047) using RT-qPCR and Western blot methods. The results revealed that the expression of Linc-ROR and FOXM1 were notably higher in TSCCA relative to that in HNOEC/HL-047 (**Figures 2A, B**). Additionally, the RT-qPCR and Western blot results indicated that expression of both Linc-ROR and FOXM1 were reduced in the TSCCA cells transfected with sh-Linc-ROR or sh-FOXM1, respectively (**Figures 2C, D**). The MTT and monoclonal formation and Transwell assay results revealed that the TSCCA cell proliferation, number of monoclonal formations as well as the invasive abilities were all significantly decreased following silencing of Linc-ROR or FOXM1, respectively (**Figures 2E–J**). Altogether, the results demonstrated that Linc-ROR and FOXM1 were highly expressed in HNSCC cells and promoted HNSCC cell proliferation and invasion.

### FOXM1 Upregulates the Expression of Linc-ROR in TSCCA Cells

**Figure 3:**
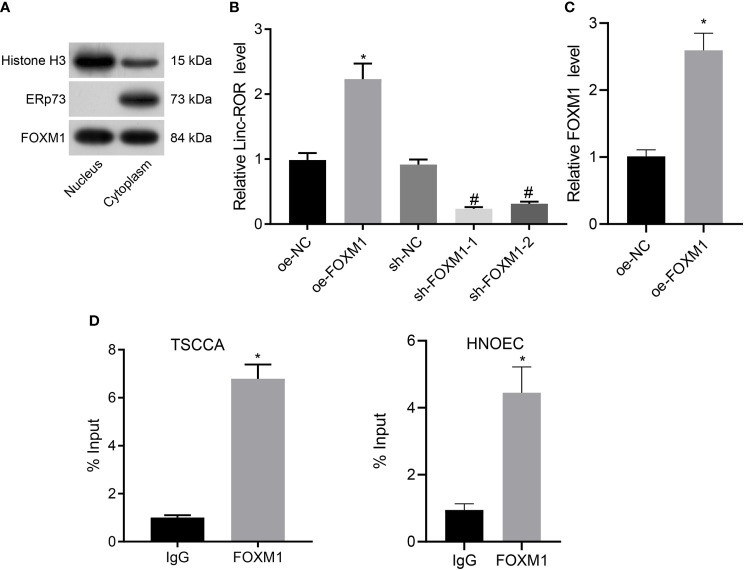
FOXM1 upregulates the expression of Linc-ROR in TSCCA cells. **(A)** Measurement of the enrichment degree of FOXM1 protein in the purified nucleus and cytoplasm. Histone H3 was considered as a nuclear marker, ERp72 was used as a cytoplasmic marker; **(B)** The expression of Linc-ROR in TSCCA cells by RT-qPCR, *p < 0.05, compared with the oe-NC group, ^#^ *p <* 0.05, compared with the sh-NC group; **(C)** Binding of FOXM1 to the promoter region of Linc-ROR in TSCCA cells determined by dual-luciferase reporter assay, *p < 0.05, compared with the oe-NC group; **(D)** The affinity of FOXM1 for the promoter of Linc-ROR in TSCCA and HNOEC/HL-047 cells determined by ChIP assay, and IgG as a negative control, *p < 0.05, compared with the IgG group. All experiments were repeated three times.

Previous literature has revealed that FOXM1 can promote the expression of Linc-ROR (12). In addition, retrieval on the USCS database suggests that FOXM1 acts as a transcription factor for Linc-ROR and binds to the promoter region of Linc-ROR (**Supplementary Figure 1**). Hence, we set out to further evaluate whether FOXM1 promoted the transcription of Linc-ROR in TSCCA cells in HNSCC. We isolated the nuclear and cytoplasmic and explored the localization of FOXM1 in TSCCA cells. FOXM1 was detected in both the nucleus and cytoplasm (**Figure 3A**). The RT-qPCR results revealed that upregulation of FOXM1 significantly promoted the expression of Linc-ROR, while the silencing of FOXM1 led to a significant inhibition of the expression in TSCCA cells (**Figure 3B**). Meanwhile, dual-luciferase reporter assay results demonstrated that upregulation of FOXM1 promoted the promoter activity of Linc-ROR in TSCCA cells (**Figure 3C**). Moreover, the results of ChIP assay demonstrated that FOXM1 bound to the Linc-ROR promoter directly in TSCCA and HNOEC/HL-047 cells (**Figure 3D**). The results suggested that FOXM1 upregulated Linc-ROR in the TSCCA cells.

### Linc-ROR Stimulates HNSCC Cell Proliferation and Invasion by Upregulating LMO4 Expression and Activating the AKT/PI3K Pathway

**Figure 4:**
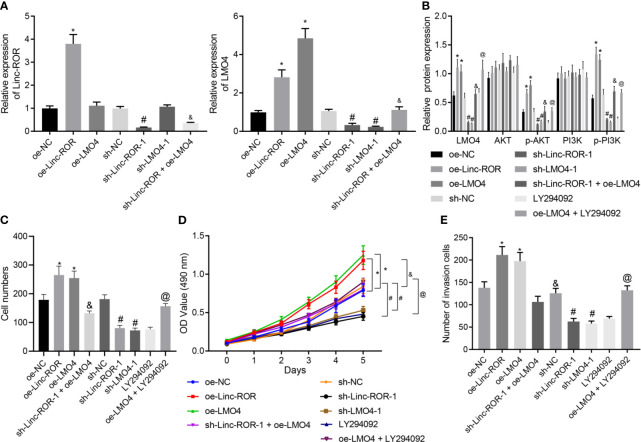
Linc-ROR promotes HNSCC cell proliferation and invasion *via* LMO4-mediated AKT/PI3K pathway activation. **(A)** The detection of Linc-ROR and LMO4 expression by RT-qPCR in TSCCA cells upon overexpression or silencing of LMO4 or Linc-ROR, sh-Linc-ROR + oe-LMO4, LY294002 or oe-LMO4 + LY294002. *p < 0.05, compared with the oe-NC group, ^#^ *p <* 0.05, compared with the sh-NC group; ^&^ *p <* 0.05, compared with the oe-LMO4 group; **(B)** Protein expression of LMO4, AKT, and PI3K along with p-AKT and p-PI3K in TSC7CA cells upon overexpression or silencing of LMO4 or Linc-ROR, sh-Linc-ROR + oe-LMO4, LY294002 or oe-LMO4 + LY294002. *p < 0.05, compared with the oe-NC group, ^#^ *p <* 0.05, compared with the sh-NC group; ^&^ *p <* 0.05, compared with the oe-LMO4 group; ^@^ *p <* 0.05, compared with the LY294002 group; **(C)** Proliferation of TSCCA cells measured by monoclonal formation assay upon overexpression or silencing of LMO4 or Linc-ROR, sh-Linc-ROR + oe-LMO4, LY294002 or oe-LMO4 + LY294002; *p < 0.05, compared with the oe-NC group, ^#^ *p <* 0.05, compared with the sh-NC group; ^&^ *p <* 0.05, compared with the oe-LMO4 group; ^@^ *p <* 0.05, compared with the LY294002 group; **(D)** Proliferation of TSCCA cells measured by MTT assay upon overexpression or silencing of LMO4 or Linc-ROR, sh-Linc-ROR + oe-LMO4, LY294002 or oe-LMO4 + LY294002. *p < 0.05, compared with the oe-NC group, ^#^ *p <* 0.05, compared with the sh-NC group; ^&^ *p <* 0.05, compared with the oe-LMO4 group; ^@^ *p <* 0.05, compared with the LY294002 group; **(E)**, Invasion of TSCCA cells measured by Transwell assay upon overexpression or silencing of LMO4 or Linc-ROR, sh-Linc-ROR + oe-LMO4, LY294002 or oe-LMO4 + LY294002. *p < 0.05, compared with the oe-NC group, ^#^ *p <* 0.05, compared with the sh-NC group. ^&^ *p <* 0.05, compared with the oe-LMO4 group; ^@^ *p <* 0.05, compared with the LY294002 group. All experiments were repeated three times.

LMO4 has been reported to perform as a facilitator of tumor development by means activating the AKT/PI3K pathway, and moreover, LMO4 was reported as a target gene of Linc-ROR (17, 23). We hypothesized that Linc-ROR regulated HNSCC cell proliferation and invasion *via* the LMO4/AKT/PI3K pathway. For verification, we treated TSCCA cells with sequences of sh-Linc-ROR-1, sh-Linc-ROR-2, sh-LMO4-1 and sh-LMO4-2 and then detected their silencing efficiency by RT-qPCR. The results illustrated a decline in the expression of Linc-ROR in response to sh-Linc-ROR-1 or sh-Linc-ROR-2. The expression of LMO4 was also found to be decreased in the presence of sh-LMO4-1 or sh-LMO4-2. sh-Linc-ROR-1 and sh-LMO4-1 exhibited superior silencing efficiency (**Supplementary Figure 2**) and were thus selected for subsequent experiments. In addition, Linc-ROR and LMO4 exhibited high levels of expression in TSCCA cells overexpressing Linc-ROR while in response to oe-LMO4, the expression of LMO4 was increased while that of Linc-ROR was not significantly altered. The expression of Linc-ROR and LMO4 was found to be decreased in TSCCA cells treated with sh-Linc-ROR and moreover, LMO4 expression was reduced while Linc-ROR was not significantly changed in the presence of LMO4 silencing. Further, Linc-ROR and LMO4 were suppressed in TSCCA cells co-treated with sh-Linc-ROR and oe-LMO4 relative to oe-LMO4 treatment alone (**Figure 4A**). After overexpressing/silencing Linc-ROR and LMO4, PI3K inhibitor LY294002 was added to the TSCCA cells, and the expression of LMO4, AKT, PI3K, and the extent of AKT and PI3K phosphorylation were detected by Western blot. The results demonstrated that the expression of LMO4 and the extent of AKT and PI3K phosphorylation were increased in the presence of overexpressed LMO4 or Linc-ROR, while an opposite trend was detected in the absence of Linc-ROR or LMO4. In addition, combined treatment with sh-Linc-ROR-1 and oe-LMO4 led to a decline of LMO4 expression and the extent of AKT and PI3K phosphorylation, which was reversed in the presence of both oe-LMO4 and LY294002 (**Figure 4B**). The MTT and monoclonal formation assay results revealed that the cell proliferation ability was enhanced and the number of monoclonal formations was significantly increased following overexpression of Linc-ROR or LMO4, which was reversed in the absence of Linc-ROR or LMO4. Linc-ROR silencing and LMO4 overexpression simultaneously reduced the proliferation and the number of monoclonal formation. Combined treatment with oe-LMO4 and LY294002 induced higher proliferation and number of monoclonal formation than LY294002 treatment alone (**Figures 4C, D**). Transwell assay findings indicated that the cell invasion was increased following overexpression of Linc-ROR or LMO4 while an opposite result was obtained in the absence of Linc-ROR or LMO4. The simultaneous silencing of Linc-ROR and overexpression of LMO4 also reduced the invasion of cells while dual treatment with oe-LMO4 and LY294002 increased cell invasion (**Figure 4E**). These findings indicated that Linc-ROR facilitated the LMO4-mediated AKT/PI3K pathway activation thus promoting HNSCC cell proliferation and invasion.

### Silencing of Linc-ROR Reverses the Promoting Effect of Over-Expression of FOXM1 on Tumor Growth *In Vivo*

**Figure 5:**
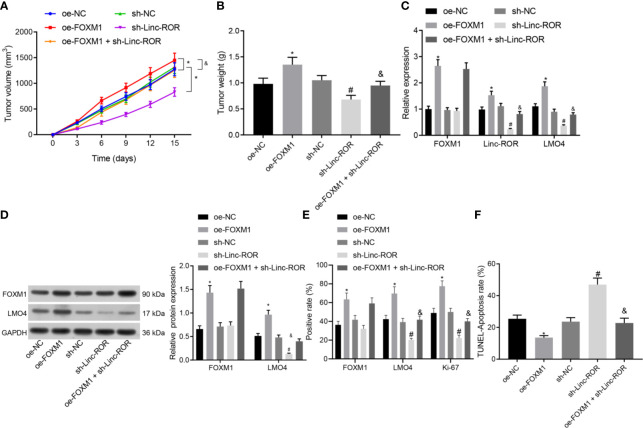
Silencing of Linc-ROR reverses the promoting effect of overexpressing FOXM1 on tumor growth *in vivo*. **(A)** Tumor growth curve; **(B)** Tumor size and weight; *p < 0.05, compared with the oe-NC group, ^#^ *p <* 0.05, compared with the sh-NC group; **(C)** Expression of FOXM1, Linc-ROR, and LMO4 in mouse tumor tissues determined by RT-qPCR; *p < 0.05, compared with the oe-NC group, ^#^ *p <* 0.05, compared with the sh-NC group; **(D)** Protein expression of FOXM1 and LMO4 in mouse tumor tissues detected by Western blot; *p < 0.05, compared with the oe-NC group, ^#^ *p <* 0.05, compared with the sh-NC group; ^&^ *p <* 0.05, compared with the oe-FOXM1 group; **(E)** The positive expression of FOXM1, LMO4, and Ki-67 proteins in mouse tumor tissues detected by IHC; **(F)** The apoptosis of cells in mouse tumor tissues detected by TUNEL assay. n = 15 for mice per group.

To further elucidate the mechanism of Linc-ROR regulating cell growth in HNSCC *in vivo*, we downregulated Linc-ROR and upregulated FOXM1 in TSCCA cells, and examined tumor growth by subcutaneous tumor formation assay. The results indicated that both tumor volume and weight were markedly elevated in the oe-FOXM1 group when compared to the oe-NC group, but decreased in the sh-Linc-ROR group compared with the sh-NC group; besides, tumor volume and weight were decreased in the oe-FOXM1 + sh-Linc-ROR group relative to the oe-FOXM1 group (**Figures 5A, B**). The RT-qPCR, Western blot and IHC results indicated that overexpression of FOXM1 upregulated the expression of Linc-ROR, LMO4, and Ki-67, while silencing of Linc-ROR downregulated the expression of LMO4 and Ki-67 as well as unchanged FOXM1 expression in the tumor tissues. The expression of FOXM1 was increased while that of Linc-ROR, LMO4, and Ki-67 was decreased in the tumor tissue of the mice in the oe-FOXM1 + sh-Linc-ROR group when compared with the oe-FOXM1 group (**Figures 5C–F**). The aforementioned results demonstrated that Linc-ROR knockdown could reverse the effects associated with the overexpression of FOXM1 which promoted tumor growth *in vivo*.

## Discussion

HNSCC remains one of the most commonly occurring and lethal malignancies known to man, characterized by a high rate of tumor recurrence as well as a low rate of survival (24). This highlights the urgent need for more effective treatment strategies for HNSCC patients. Both our *in vitro* and *in vivo* experimental results highlighted the potential of FOXM1 to stimulate the occurrence and progression of HNSCC *via* Linc-ROR promotion and subsequent activation of the LMO4-dependent AKT/PI3K signaling pathway.

FOXM1 and Linc-ROR were found to be abundant in both the clinical samples of HNSCC patients as well as the cell lines. Additionally, evidence was obtained implicating these two factors in the acceleration of HNSCC cell invasion and migration. Similarly, FOXM1 has been reported to exhibit high levels of expression in tongue squamous cell carcinoma, illustrated by its contribution to the stimulation of the migration and invasion capacities of SCC9 and SCC25 cells (25). The overexpression of FOXM1 has been shown to promote cell proliferation and migration while acting to inhibit the apoptosis of hypopharyngeal squamous cell carcinoma, ultimately contributing to poor clinical prognosis (26). Linc-ROR has been highlighted to promote tumorigenesis by decoying gene-specific histone methylation (23). High levels of Linc-ROR were identified in oral squamous cell carcinomas tissues, often leading to poor treatment response and tumor recurrence (14).

Linc-ROR could be transcriptionally activated by FOXM1. The upregulation of Linc-ROR and FOXM1 has been reported to impair the sensitivity of hepatocellular carcinoma cells to sorafenib (12). The aforementioned report suggests a positive correlation between the expression of FOXM1 and the expression of Linc-ROR. Consistently, the results of our study revealed that FOXM1 could upregulate the expression of Linc-ROR in HNSCC cells, while the knockdown of Linc-ROR was shown to normalize the stimulatory effect associated with FOXM1 on tumor growth in the xenograft model of HNSCC cells. Since little progress has been made in the FOXM1/Linc-ROR signaling in the process of HNSCC, additional studies are still warranted to validate these findings.

Another key observation of the current study indicated that Linc-ROR could augment the expression of LMO4, leading to an increase in the activity of the AKT/PI3K signaling pathway. A recent study demonstrated that forced LMO4 expression could counteract breast cancer cell cycle redistribution and inhibit cell migration *in vitro* alongside tumor growth inhibition *in vivo* induced by siRNA-mediated lncRNA SNHG1 knockdown (15). Consistent with the findings of the current study, the expression of LMO4 has been reported to decrease in cells following the knockdown of Linc-ROR (23). Abundant levels of LMO4 have been highlighted in HNSCC cell lines with studies suggesting its contributory role in the metastasis, proliferation, and angiogenesis of HNSCC (16). In addition, LMO4 possesses the capacity to potentiate the proliferation and invasion of gastric cancer cells by means of activating the AKT/PI3K signaling pathway (27). Targeting the AKT/PI3K signaling pathway has been proposed as an effective therapeutic approach for patients with HNSCC since its suppression has been shown to trigger cell cycle arrest and apoptosis (28). Additionally, the crucial roles played by lncRNAs in connection with the AKT/PI3K signaling pathway have been widely documented in a numerous human cancers (29, 30). Linc-ROR has been identified as an upstream modulator or a downstream effector of major signaling pathways influencing endometrial cancer metastasis, including the AKT/PI3K signaling pathway (31). Linc-ROR has been demonstrated to trigger arsenite-transformed keratinocyte proliferation by inhibiting p53 activity and activating the AKT/PI3K signaling pathway (32). Mutations to the tumor-suppressor gene p53 represent one of the most common genomic alterations that take place in HNSCC with the mutational profile of p53 has been highlighted as an independent prognostic factor for HNSCC (33). Jin et al. proposed that p53 can directly bind to the lincRNA-p21 promoter and inhibit its expression, thus down-regulating the JAK2/STAT3 signaling pathway and subsequently inhibiting the progression of HNSCC (34). The activation of FOXM1 as well as the AKT/PI3K signaling pathway has been reported to aid in driving hepatic stellate cell activation as well as liver fibrosis (35). Based on the aforementioned information, we are further convinced that the up-regulation of FOXM1 functions as a promoter of HNSCC by means of regulating the Linc-ROR/LMO4/AKT/PI3K axis.

## Conclusion

**Figure 6:**
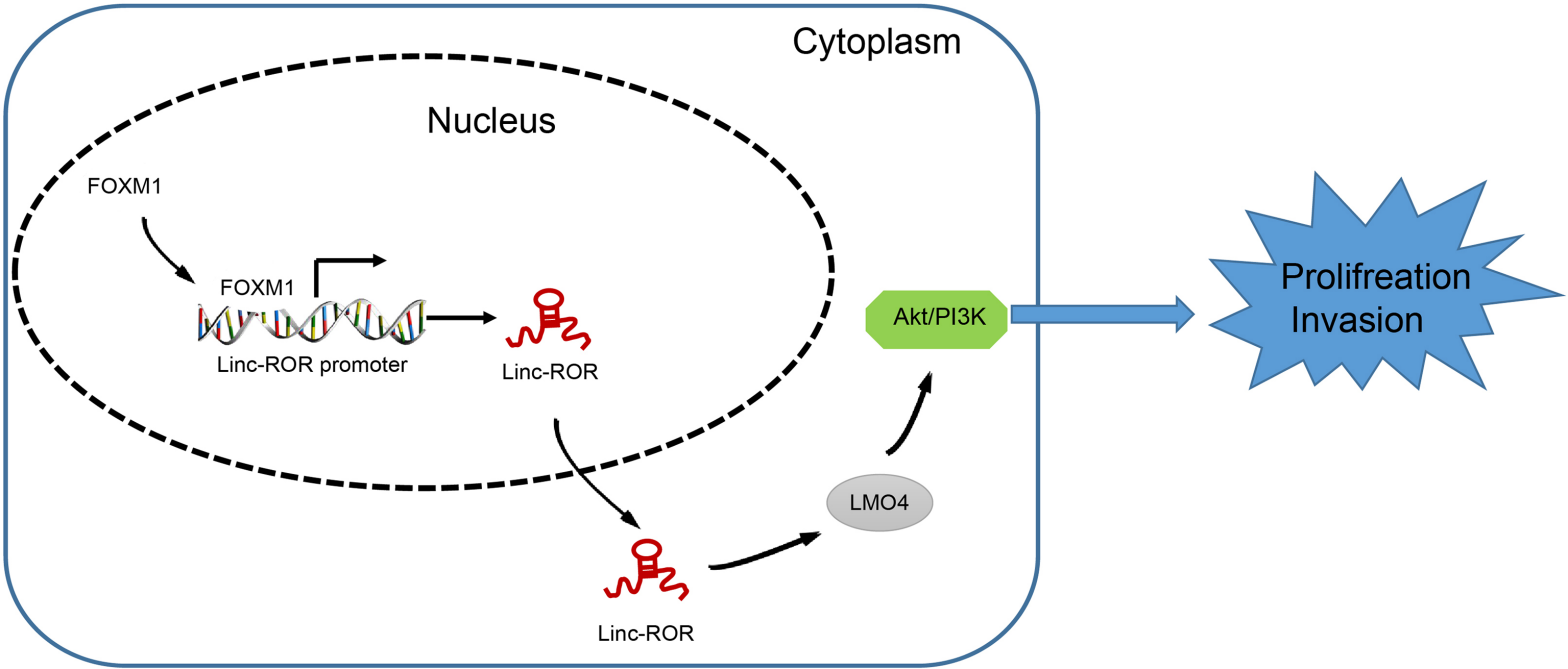
The mechanism of FOXM1 unveiled in the present study may provide an insight for the development of new targets for HNSCC.

In conclusion, the key observations of our study highlight a novel mechanism by which FOXM1 serves as an oncogenic factor in the tumorigenesis of HNSCC *via* regulation of the Linc-ROR/LMO4/AKT/PI3K signaling cascade. The functional mechanism of FOXM1 unveiled in the present study may provide valuable insight for the development of new targets for HNSCC (**Figure 6**). Further investigations into the interaction between FOXM1, Linc-ROR, LMO4 and the AKT/PI3K signaling pathway are still required to further understand and elucidate the specific mechanisms associated with FOXM1 in HNSCC, so as to validate its applicable value from a clinical practice perspective.

## Data Availability Statement

The original contributions presented in the study are included in the article/**Supplementary Material**. Further inquiries can be directed to the corresponding author.

## Ethics Statement

The studies involving human participants were reviewed and approved by the Ethics Committee of Peking University First Hospital. The patients/participants provided their written informed consent to participate in this study. The animal study was reviewed and approved by the Ethics Committee of Peking University First Hospital.

## Author Contributions

XM and HZ wrote the paper. QL and ES conceived the experiments. YQ and SX analyzed the data. TL collected and provided the sample for this study. All authors contributed to the article and approved the submitted version.

## Conflict of Interest

The authors declare that the research was conducted in the absence of any commercial or financial relationships that could be construed as a potential conflict of interest.

## Publisher’s Note

All claims expressed in this article are solely those of the authors and do not necessarily represent those of their affiliated organizations, or those of the publisher, the editors and the reviewers. Any product that may be evaluated in this article, or claim that may be made by its manufacturer, is not guaranteed or endorsed by the publisher.

## Supplementary Material

Supplementary Figure 1 FOXM1 bound to the promoter of Linc-ROR according to the UCSC genome database (gene assembly version hg19, DNA range: chr18: 54,729,703-54,747,249).

Supplementary Figure 2 Silencing efficiency of sh-Linc-ROR-1, sh-Linc-ROR-2, sh-LMO4-1 and sh-LMO4-2 determined by RT-qPCR. **p <* 0.05, compared with the sh-NC group.
