# Supplementary figures and images for: FOXM1 Promotes Head and Neck Squamous Cell Carcinoma via Activation of the Linc-ROR/LMO4/AKT/PI3K Axis
Source: Front Oncol. 2021 Aug 10;11:658712. doi: 10.3389/fonc.2021.658712 (PMC8383294; doi:10.3389/fonc.2021.658712)

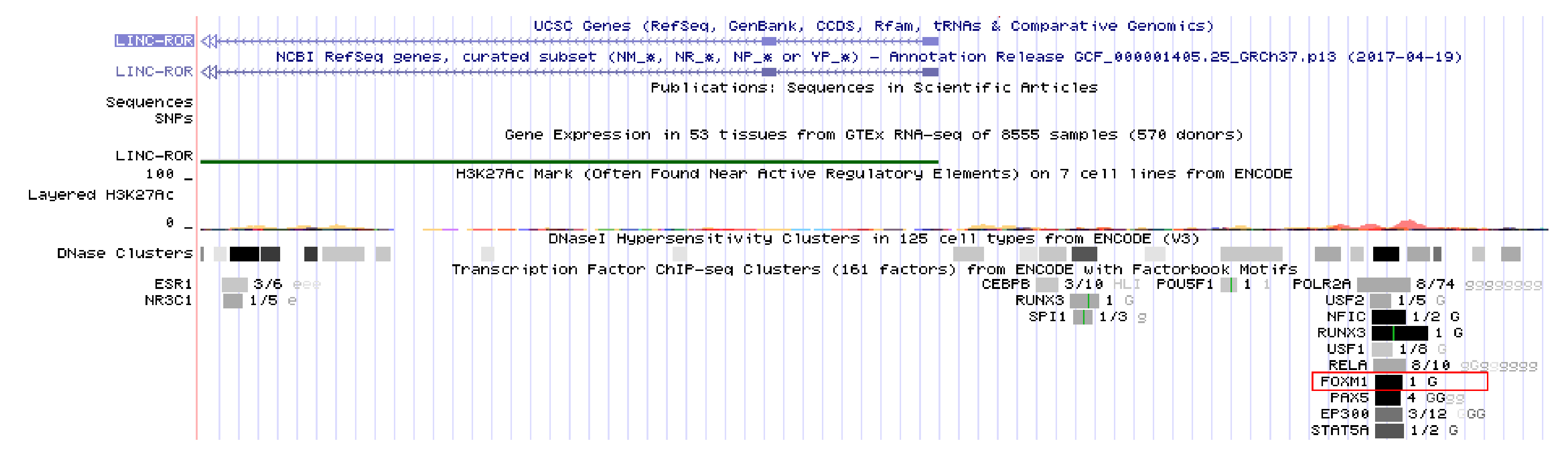

Supplement: Supplementary Figure 1 — FOXM1 bound to the promoter of Linc-ROR according to the UCSC genome database (gene assembly version hg19, DNA range: chr18: 54,729,703-54,747,249). [file Image_1.jpeg]

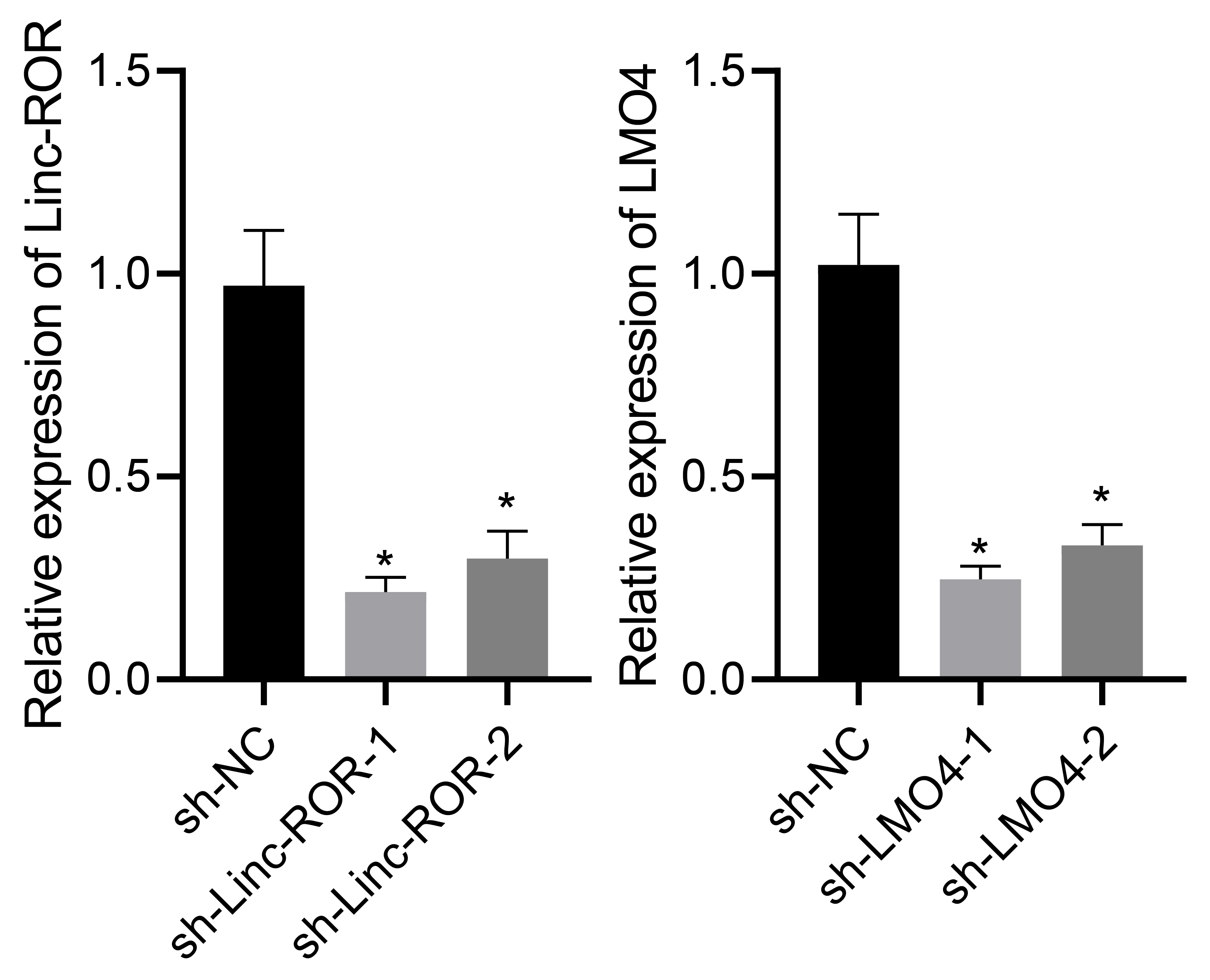

Supplement: Supplementary Figure 2 — Silencing efficiency of sh-Linc-ROR-1, sh-Linc-ROR-2, sh-LMO4-1 and sh-LMO4-2 determined by RT-qPCR. *p < 0.05, compared with the sh-NC group. [file Image_2.jpeg]
