# Supplementary material for: FOXM1 Promotes Head and Neck Squamous Cell Carcinoma via Activation of the Linc-ROR/LMO4/AKT/PI3K Axis
Source: Front Oncol. 2021 Aug 10;11:658712. doi: 10.3389/fonc.2021.658712 (PMC8383294; doi:10.3389/fonc.2021.658712)
Supplement: Supplementary file 3 [file Table_1.docx]

**Table S1** Primer sequences for RT-qPCR

| Gene | Primer sequences |
| --- | --- |
| Linc-ROR (human) | Forward: 5’-ATCAGCAGGTCTCAGGGTTG -3’ |
|  | Reverse: 5’- GGTTCTAAGCAGAGTGGCGA -3’ |
| FOXM1 | Forward: 5’-GGAGGAAATGCCACACTTAGCG-3’ |
|  | Reverse: 5’-TAGGACTTCTTGGGTCTTGGGGTG-3’ |
| LMO4 | Forward: 5’- GCAAGCGCAGGAAAGTTGAG -3’ |
|  | Reverse: 5’- GCTGTGCCAATAGCTGTCCA -3’ |
| GAPDH | Forward: 5’- GACAGTCAGCCGCATCTTCT -3’ |
|  | Reverse: 5’- GCGCCCAATACGACCAAATC -3’ |
